# Pelvic Inflammatory Disease With Presumptive Tubo‐Ovarian Abscess Presenting With Rectal Spasm

**DOI:** 10.1155/crog/4690633

**Published:** 2026-03-03

**Authors:** Michael C. Larkins, Ariel L. Lanier, Ciara Smith

**Affiliations:** ^1^ Department of Emergency Medicine, Boonshoft School of Medicine, Wright State University, Fairborn, Ohio, USA, wright.edu; ^2^ Department of Obstetrics and Gynecology, Boonshoft School of Medicine, Wright State University, Dayton, Ohio, USA, wright.edu

## Abstract

**Background:**

Pelvic inflammatory disease (PID) is an infectious process of the upper female genital tract, commonly caused by *Chlamydia trachomatis*, *Neisseria gonorrhoeae*, or *Mycoplasma genitalium*. Complications may include tubo‐ovarian abscess (TOA) due to localized infection. Typical symptoms include pelvic pain, vaginal discharge, dyspareunia, and abnormal bleeding, with diagnosis often made clinically and supported by imaging and laboratory testing. Empiric antibiotic therapy is recommended promptly to prevent long‐term sequelae. Rectal spasms, or proctalgia fugax, are characterized by fleeting anorectal pain episodes and are not well described in association with PID or TOA.

**Case:**

A 43‐year‐old female G12P5065 with noninsulin dependent diabetes presented with severe, spasmodic rectal pain and a 1‐week history of lower abdominal discomfort with vaginal discharge and malodor. She had recently been prescribed metronidazole for presumed bacterial vaginosis but had not initiated therapy. Examination revealed foul‐smelling vaginal fluid, an erythematous cervix with cervical motion tenderness, and active anal sphincter spasm without palpable masses or fluctuance. Laboratory studies showed leukocytosis (16.9 k/uL); imaging revealed right ovarian soft tissue thickening abutting pelvic structures concerning for PID/TOA. Transvaginal ultrasound demonstrated benign right ovarian cysts. STI panel was positive for *C. trachomatis*, *Trichomonas vaginalis*, and bacterial vaginosis. Empiric therapy with ceftriaxone, doxycycline, and metronidazole was initiated. The patient was admitted for pain control, including methocarbamol for rectal spasms, and discharged after clinical improvement with outpatient follow‐up.

**Conclusion:**

This case highlights atypical presentation of PID with severe rectal spasms, likely secondary to local inflammation adjacent to the rectum. While PID commonly presents with pelvic pain and vaginal symptoms, clinicians should maintain a broad differential for rectal pain and consider pelvic pathology when initial rectal evaluation is unrevealing. Prompt examination, imaging, empiric therapy, and specialist consultation remain critical in managing complicated PID presentations.

## 1. Introduction

Pelvic inflammatory disease (PID) is defined as an infectious inflammation of the upper genital tract in female patients. [1] Typically this occurs as an ascending infection from the lower genital tract, with the most common causal agents including *Neisseria gonorrhoeae*, *Chlamydia trachomatis*, and *Mycoplasma genitalium*. Coinfections with other species such as *Streptococcus* species, *Escherichia coli*, and so on, are also common [2]. Tubo‐ovarian abscess (TOA) as well as pelvic abscess can occur as complications due to localized containment of the infection. Patients typically present with lower abdominal or pelvic pain, vaginal discharge, dyspareunia, abnormal vaginal bleeding, and may report urinary symptoms. The clinical diagnosis of PID is made when cervical, uterine, or adnexal tenderness is noted during speculum or bimanual exam [3]. Other associated findings include cervical discharge or friability, fever, and positive causative agent testing. PID is the most common gynecological reason for admission in US hospitals, and can lead to complications including infertility, chronic pain, and subsequent ectopic pregnancies [4]. While spontaneous resolution can occur, PID/TOA are primarily clinical diagnoses, and empiric treatment with antibiotics is indicated to prevent sequelae.

Rectal spasms are not commonly associated with PID or TOA; to date no literature has linked the two. Given the proximity of the female reproductive tract and distal gastrointestinal tract, adjacent involvement of one to the other is not implausible. It has been reported in the literature that patients with chronic pain of the levator ani muscle can present with vaginal pain, given the closeness of these structures [5]. We report a case of PID with presumptive TOA and inflammation‐associated rectal spasms, possibly in line with a diagnosis of proctalgia fugax, a disorder characterized by recurring episodes of rectal pain on the spectrum of functional gastrointestinal disorders [6]. This is the first such connection reported between these two conditions to date.

## 2. Case Presentation

A 43‐year‐old female, G12P5065, with a history of noninsulin‐dependent diabetes mellitus presented to the emergency department (ED) with severe rectal pain described as spasmodic in nature. She reported no fever, chills, nausea, vomiting, vaginal bleeding, or urinary symptoms and denied previous uterine prolapse. She presented hemodynamically stable and afebrile.

On physical exam no abdominal tenderness was appreciated though the abdomen was significantly obese. Rectal exam demonstrated scant, hemoccult‐negative stool in the rectal vault though active anal sphincter spasm could be appreciated. No masses, discharge, rash, or fluctuance were appreciated. Speculum exam demonstrated white/yellow foul‐smelling fluid in the vaginal canal with an erythematous cervix. Cervical motion tenderness was appreciated.

Initial workup consisted of a complete blood count, basic metabolic panel, hepatic function panel, lipase, urinalysis, urine pregnancy screen, and culture of the vaginal fluid. The patient was treated with IV fluids, ondansetron, and morphine for symptomatic relief in the interim. Workup was notable for leukocytosis of 16.9 k/*μ*L; urinalysis demonstrated 6‐10 WBC′s and 11–25 squamous epithelial cells, concerning for contamination.

**Figure 1:**
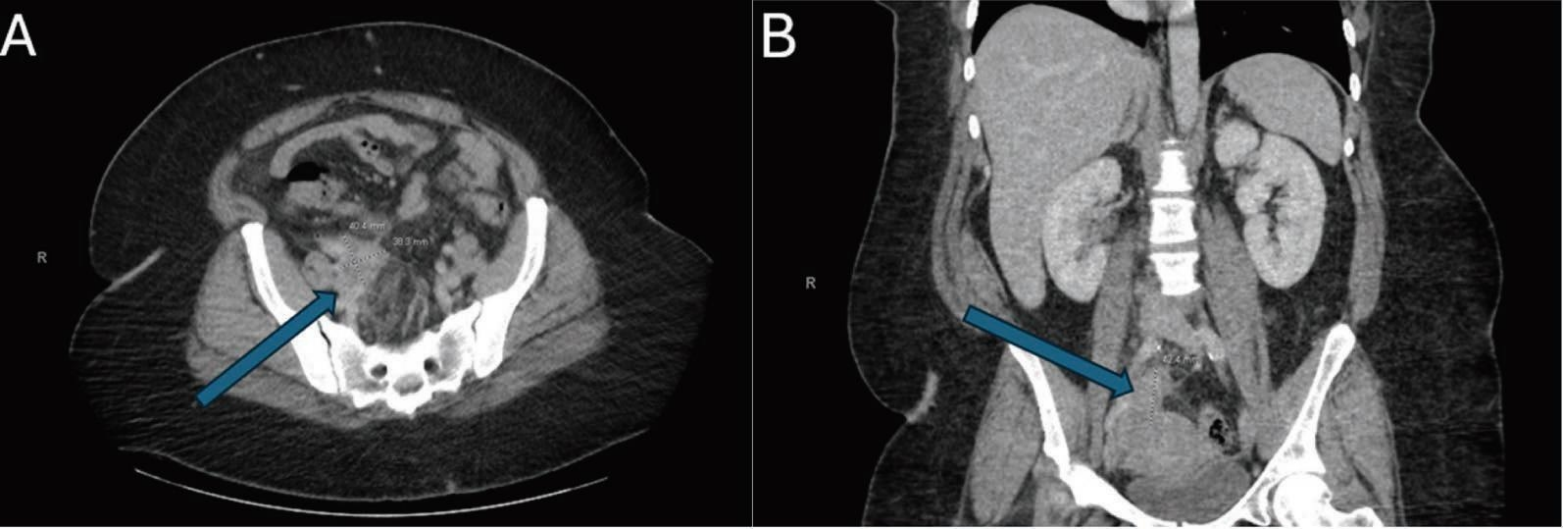
Computed tomography (CT) of the abdomen and pelvis with soft tissue thickening adjacent to the colon with (A) axial and (B) coronal slices from a CT abdomen pelvis with intravenous contrast demonstrating a 4 cm × 4 cm area of soft tissue thickening adjacent to the uterine fundus, colon, cecum, and terminal ileum concerning for pelvic inflammatory disease (blue arrows point to this area on both panels). A tubo‐ovarian abscess could not be excluded.

CT abdomen pelvis with IV contrast demonstrated a 4 cm area of soft tissue thickening rising from the right ovary that abutted the uterine fundus, colon, cecum, and terminal ileum concerning for pelvic PID (see Figure 1A,B). A TOA could not be fully excluded. A transvaginal ultrasound demonstrated two right ovarian cysts with benign features. The obstetrics and gynecology (OB/GYN) service was consulted, and empiric antibiotic therapy was initiated with ceftriaxone, doxycycline, and metronidazole. Subsequent vaginal testing returned positive for *C. trachomatis*, bacterial vaginosis, and *Trichomonas vaginalis*. Swabs from the rectum were negative for similar infections.

Following evaluation, the patient was admitted to the OB/GYN service and treated with methocarbamol for her persistent rectal spasms in addition to scheduled acetaminophen and ibuprofen. She was discharged 1 day later with close outpatient follow‐up after her symptoms were controlled and her rectal spasms abated.

## 3. Discussion

The diagnosis of PID/TOA is primarily clinical, though laboratory evaluation should include pregnancy testing to exclude the possibility of ectopic pregnancy [1]. Testing for causative sexually‐transmitted diseases and other vaginal infectious agents should also be performed. Recent literature also mentions the use of cancer antigen (CA)‐125, traditionally a test that may indicate a patient has a gynecologic cancer. [7] Elevated serum levels are associated with failure of conservative management of TOA, though it is not yet considered a tool for diagnosing TOA. Pelvic ultrasound can be considered to assess for potential adnexal mass though absence of findings does not exclude PID or TOA [1]. CT imaging can assist in both evaluating the severity of infection in patients with high suspicion for PID/TOA, and can also evaluate for other etiologies for patients presenting with pelvic, vaginal, or rectal pain [8]. This may be especially beneficial in patients with unusual or atypical causes of PID/TOA, such as actinomycosis, and magnetic resonance can also be considered [9]. Treatment consists of empiric antibiotics, including ceftriaxone, cefotetan, or cefoxitin, with doxycycline [10]. If the patient is treated with ceftriaxone, metronidazole should be added for enhanced anaerobic coverage. Given the significant risk of sequelae including sterility, ectopic pregnancy, and possibility of deterioration to sepsis, prompt treatment following clinical diagnosis is necessary [4]. Inflammatory markers were not obtained during our evaluation of this patient nor were they thought to be diagnostic, as they would likely have been elevated secondary to the patient′s infection, and would not have been useful in diagnosing a disease of noninfectious etiology. Furthermore, discharge after 1 day of admission is somewhat atypical for a patient with a TOA. It was felt that given the patient had normal vitals, had good pain control on her inpatient regimen, and was comfortable following up outpatient, that she was appropriate to discharge with close outpatient follow up. No definitive drainable abscess was noted, so drainage or total abdominal hysterectomy with bilateral salpingo‐oophorectomy was not considered necessary. Some sources do report transition to oral antibiotics, and discharge after 24–48 h of inpatient observation is appropriate, though this is specific to each patient [11].

The authors conducted a literature review for presentations of PID or tubo‐ovarian abscess presenting with rectal spasms on both PubMed and Google Scholar using the following searches: “pelvic inflammatory disease” and “rectal spasm”; “tubo‐ovarian abscess” and “rectal spasm”; “pelvic inflammatory disease” and “rectal pain”; “tubo‐ovarian abscess” and “rectal pain.” Permutations of tubo‐ovarian without spacing or a dash between “tubo” and “ovarian” were also checked. Dunphy et al. reported levator ani syndrome presenting with vaginal pain, a related but not exact match to what the authors report in this case report [5]. Tou et al. report a colo‐ovarian fistula that mimicked a tubo‐ovarian abscess but again did not present with rectal pain or spasms and was not found to be a TOA [12]. Arda et al. report a sexually inactive adolescent who presented with fever and abdominal pain who was found to have a firm, tender mass on rectal examination [13]. This report likely describes a similar mechanism to the one we present in this case report, in that a localized infection caused localized inflammatory changes adjacent to the intestine, though the patient report in Tou et al. did ultimately require laparoscopy and was found to have *E. coli* as the causative agent, as opposed to *C. trachomatis*, bacterial vaginosis, and *T. vaginalis* as we report in our case. Finally, Chow and Chen reported in 2000 a case series on two patients found to have TOA mimicking malignancy, resulting in laparoscopy, which revealed chronic abscesses with associated omental abscess [14]. These patients presented with tenesmus, though one of which was ultimately found to grow Viridans streptococci on culture and both had dense fibrotic adhesions within the abdomen. While similar to what we report in our case, the patient presented did not appear to have chronic TOA and ultimately did not require laparoscopy, nor was potential malignancy considered a likely diagnosis.

The differential for rectal spasms includes anorectal disorders such as anal fissure, rectal foreign body, Crohn′s disease, anal sexually‐transmitted disease, rectal abscess, pilonidal cyst, parasitic infection, hemorrhoids (internal versus external, considering thrombosed or strangulated hemorrhoids as well), constipation, strangulated rectal prolapse, obstructing rectal cancer, and proctitis [15, 16]. Given the broad range in potential etiologies for this patient′s presentation, a comprehensive work up including pelvic exam and CT imaging was necessary, as several potential diagnoses are life‐threatening in nature. Less commonly and underreported in the literature, given the close proximity of the vaginal canal and pelvic reproductive organs, infections/pathology involving these organs systems should also be considered. As far back as the mid‐1950′s discussion has been had of extra rectal causes of rectal pain/spasm, and localized inflammation and mass effect from infection adjacent to the anus/rectum can induce pain and spasms without infection truly being present within the rectum [17].

Proctalgia fugax, another related disorder, is defined by the Rome III diagnostic criteria as episodes of fleeting pain that recurs over weeks, which is localized to the anus or lower rectum and that lasts seconds to minutes with complete resolution between [6]. It is a diagnosis of exclusion, though given the muscular nature of the patient′s chief complaint, this diagnosis was heavily considered by the inpatient team. The patient described in this case report did experience symptomatic improvement with the addition of a muscle relaxer, strengthening the possibility that this patient had associated proctalgia fugax, though a definitive diagnosis could not be made in the short hospital course this patient experienced.

We also did consider other related differentials, such as levator ani syndrome, proctitis, and neuropathic pain [5]. Levator ani syndrome, generally a diagnosis of exclusion related to pelvic floor muscle spasm and elevated anal resting pressure, requires exclusion of other causes of rectal pain, such as localized infection or adjacent abscess. We did not perform a comprehensive examination of the pelvic floor muscles or obtain a pelvic/rectal MRI for further evaluation of noninfectious etiologies. Formal diagnosis of levator ani syndrome, for instance, would require a pelvic MRI demonstrating obvious spasm or atrophy of the anal sphincter; furthermore, magnetic resonance defecography can be used to evaluate for pelvic floor dysfunction in real time. In a similar vein, proctitis, localized inflammation of the rectum, could present similarly, with rectal spasms as this patient presented. Causes of proctitis can include inflammatory bowel disease, localized infection, anal receptive intercourse, and radiotherapy. This patient did not report any anal receptive intercourse or history of radiotherapy. While colonoscopy was not performed, no infectious involvement of the anus/rectum was noted during the physical exam or the CT the patient underwent. Her improvement with antibiotics and muscle relaxers also helps reinforce the diagnosis being of an infectious etiology. Finally, neuropathic pain could also be considered, particularly since this patient had an elevated A1c (8.9). Given the localized infection seen on CT, this seemed less likely, though neuropathy has been found to play into both chronic rectal pain and levator ani syndrome, among other related anal pain disorders.

One complicating factor of this case was the patient′s body habitus, which made her abdominal exam likely less useful in assessing the location of her pain. Her body mass index was 37.7, and the obesity of her abdomen may have contributed to the abdominal exam, which lacked tenderness to any quadrant or the pelvic region. Additionally, other pelvic pathology was noted, mainly two benign ovarian cysts (seen on United States). It was felt that given no sign of ovarian torsion on either scan, the obvious area of infection adjacent to the rectum, and no sign of hemorrhage/complexity regarding her cysts, that the cysts were not relevant and the source of her pain was the inflamed area concerning for PID and likely presumptive TOA. The patient′s pelvic exam subsequently supports this assertion.

## 4. Conclusion

This case highlights the importance of considering a broad differential with regards to patients presenting with rectal pain. Consideration for abnormal presentations and thorough evaluation in the ED is vital. Speculum examination in appropriate patients should be executed both for evaluation of female patients with rectal symptoms if a high degree of suspicion is present and rectal examination is not conclusive. It is thought that the inflammation and infection adjacent to the colon of the patient in this case contributed to, if not directly induced, the rectal spasms observed on presentation. In cases of advanced PID or TOA, localized inflammation and mass effect from infected adnexal structures can irritate adjacent pelvic organs, including the rectum, leading to symptoms such as rectal pain, tenesmus, or spasm.

## Funding

No funding was received for this manuscript.

## Disclosure

The views and opinions expressed in this article/presentation are those of the author(s) and do not necessarily reflect official policy or position of the United States Air Force, Defense Health Agency, Department of Defense, or U.S. Government.

## Ethics Statement

In our institute, ethical approval is exempt, depending on acquired patient consent.

## Consent

Written informed consent was obtained from the patient per institutional policy to publish this case report.

## Conflicts of Interest

The author declares no conflicts of interest.

## Data Availability

Data sharing is not applicable to this article as no datasets were generated or analyzed during the current study.
